# Preliminary report of an autochthonous chikungunya outbreak in France, July to September 2017

**DOI:** 10.2807/1560-7917.ES.2017.22.39.17-00647

**Published:** 2017-09-28

**Authors:** Clémentine Calba, Mathilde Guerbois-Galla, Florian Franke, Charles Jeannin, Michelle Auzet-Caillaud, Gilda Grard, Lucette Pigaglio, Anne Decoppet, Joel Weicherding, Marie-Christine Savaill, Manuel Munoz-Riviero, Pascal Chaud, Bernard Cadiou, Lauriane Ramalli, Pierre Fournier, Harold Noël, Xavier De Lamballerie, Marie-Claire Paty, Isabelle Leparc-Goffart

**Affiliations:** 1 Regional office of the French National Public Health Agency (Cire Paca-Corse), Marseille, France; 2 Armed Forces Biomedical Research Institute, National Reference Laboratory for arboviruses, Marseille, France; 3 UMR EPV ‘Émergence des Pathologies Virales’, Aix-Marseille University - IRD 190 - Inserm 1207 – EHESP – IHU Méditerranée Infection, Marseille, France; 4 Entente Interdépartementale pour la Démoustication du littoral Méditerranéen (EID Méditerranée), Public mosquito control operator, Montpellier, France; 5 Regional Health Agency of Provence-Alpes-Côte d’Azur (ARS Paca), Toulon, France; 6 Regional Health Agency of Provence-Alpes-Côte d’Azur (ARS Paca), Marseille, France; 7 Entente Interdépartementale pour la Démoustication du littoral Méditerranéen (EID Méditerranée), Public mosquito control operator, Le Cannet-des-Maures, France; 8 European Programme for Intervention Epidemiology Training (EPIET), European Centre for Disease Prevention and Control (ECDC), Stockholm, Sweden; 9 Eurofins Biomnis, Lyon, France; 10 French National Public Health Agency (Santé publique France), Saint-Maurice, France

**Keywords:** Chikungunya virus, autochthonous outbreak, ECSA lineage, France, CHIKV, *Aedes albopictus*

## Abstract

In August 2017, an autochthonous chikungunya case was reported in south-east France. By mid-September, eight additional autochthonous cases were found in the index case’s neighbourhood, where the chikungunya virus vector *Aedes albopictus* was observed. Genomic characterisation identified an East-Central South African (ECSA) lineage strain, probably from the Central African region and carrying an adaptive mutation facilitating transmission by *Ae. albopictus*. The event confirms we need early case detection and response to contain chikungunya in Europe.

On 9 August 2017, Eurofins Biomnis laboratory in Lyon, notified to the French Regional Health Authority a chikungunya virus (CHIKV)-positive test result by real-time reverse transcription PCR (RT-PCR) in a man who had not travelled abroad in the 15 days before onset of symptoms. The National Reference Laboratory (NRL) for arboviruses confirmed the case on 11 August.

This index case lived in a self-contained house in a residential area in Le Cannet-des-Maures. He reported having developed high fever on 2 August, along with strong and incapacitating joint pains in the wrists and fingers, and oedema of extremities. Le Cannet-des-Maures has 4,500 inhabitants and is located in the department of Var in the south-east of France (Figure 1).

**Figure 1:**
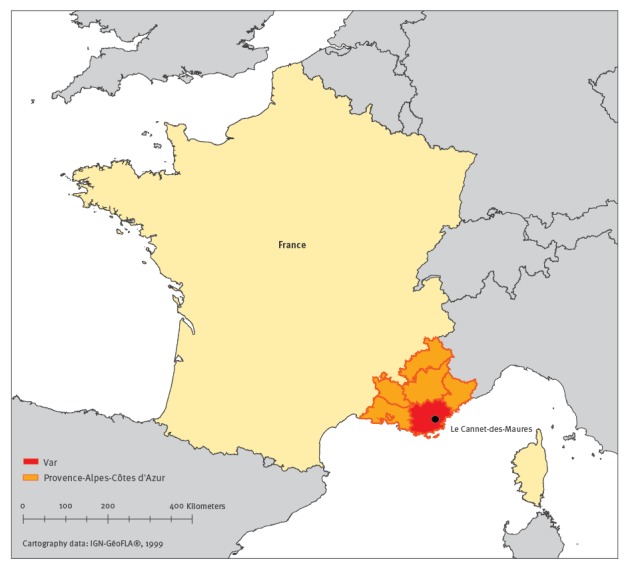
Map with geographical location of Le Cannet-des-Maures, department of Var, south-east France

The national and regional health authorities initiated a multidisciplinary investigation to determine the source of infection and the extent of CHIKV dissemination.

## Epidemiological investigations and laboratory analyses

For the epidemiological investigation, we defined an autochthonous case as a person without a history of foreign travel within 15 days prior to symptoms onset. A suspected case of chikungunya was defined by sudden onset of fever (≥ 38.5 °C) and arthralgia, not explained by another medical condition. Cases were classified as probable and confirmed according to laboratory criteria: (i) a probable case presented a single serology positive for IgM; (ii) a confirmed case presented a positive RT-PCR.

We conducted a door-to-door case-finding campaign on 14 and 17 August in all households within a 200 m radius around the index case’s home in Le Cannet-des-Maures to identify and test any person with symptoms compatible with CHIKV infection, among households’ members and visitors.

We also contacted general practitioners (GP), medical laboratories and emergency services in the municipality and its surroundings, by phone and email, to request them to report as quickly as possible any patient with symptoms compatible with CHIKV infection since 1 June.

In parallel, we reviewed the French arbovirus diseases surveillance database to look for a possible primary case among the imported chikungunya cases at local and national level.

For each suspected case identified, blood samples were collected and analysed for evidence of chikungunya infection by RT-PCR and/or serology. For IgM detection, the NRL used an in-house IgM antibody-capture enzyme-linked immunoabsorbent assay (MAC-ELISA) and for IgG indirect ELISA, with precipitated and inactivated virus produced in cell culture as the antigen. Molecular characterisation was conducted by the NRL directly from the index case's serum, using Next Generation Sequencing as previously reported [1]. Sequence data were used for phylogenetic reconstruction.

### Entomological investigations

The Entente Interdépartementale de Démoustication Méditerranée, the vector control operators, carried out investigations inside and around the index case’s home and workplace as soon as the case was identified.

Furthermore, entomological investigations were carried out for each of the additional autochthonous cases. Adult mosquito traps (13 BG Sentinel and 13 BG-gravid *Aedes* trap (GAT)) were placed in several gardens in the treated area in order to estimate vector densities, as an indicator of the effectiveness of the control measures.

### Epidemiological and laboratory results

**Figure 2:**
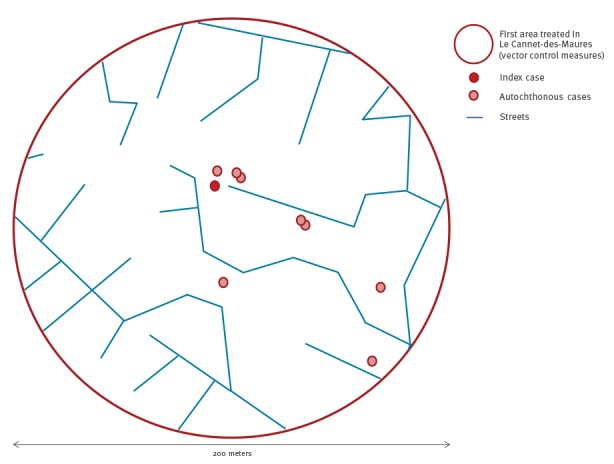
Map of the chikungunya virus circulation area following the detection of the index case and further autochthonous cases, Le Cannet-des-Maures, France, 28 July–14 September 2017 (n=9 cases^a^)

^a^ Seven laboratory-confirmed and two probable.

**Figure 3:**
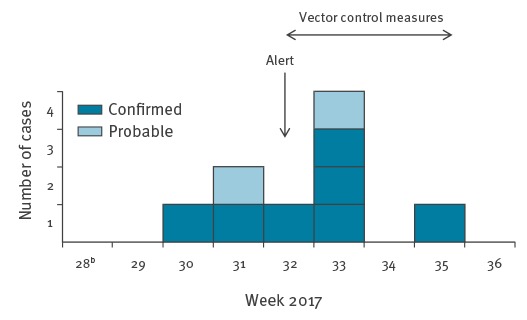
Epidemic curve of autochthonous chikungunya cases by week of symptom onset, Le Cannet-des-Maures, France, July–14 September 2017 (n=9 cases^a^)

^a^ Seven laboratory-confirmed and two probable.

^b^ Week 28 starting on 10 July 2017.

The active case finding led to the identification of additional suspected autochthonous cases. By 14 September, seven cases (including the index case) were laboratory-confirmed based on CHIKV genome detection by real-time RT-PCR and two were classified as probable based on serological evidence. Seven were men and two women, aged 33 to 77 years. Cases resided within a 200 m radius around the index case’s home (Figure 2) and reported onset of symptoms between 28 July and 30 August (Figure 3). The most common clinical symptoms were arthralgia (8/9), asthenia (8/9), fever higher than 38.5°C (7/9), headache (6/9) and skin rash (5/9).

During the door-to-door investigations, several persons reported a history of recent travel to subtropical or tropical areas but none of them developed symptoms compatible with chikungunya. Since the beginning of the year 2017, no imported chikungunya case has been reported in the region and the two imported cases, returning from Brazil in May and June, respectively, identified at the national level, did not travel to this part of France.

**Figure 4:**
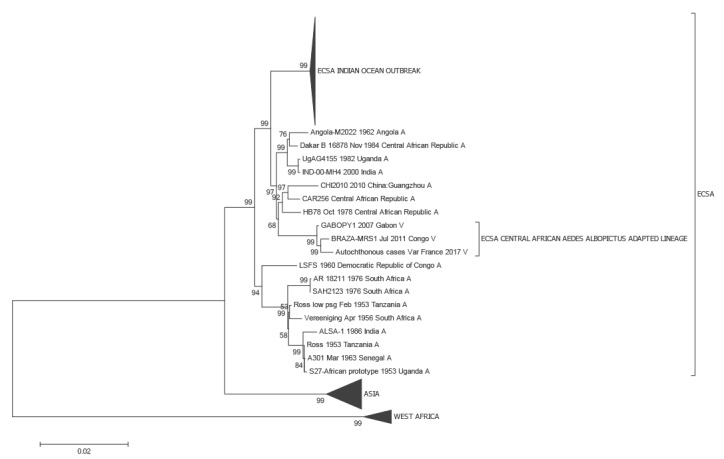
Phylogenetic relations between the Var France 2017 chikungunya virus and other chikungunya viruses

Virus genomic information was obtained from the index case only. Next Generation Sequencing performed directly from nucleic acids extracted from the serum allowed to reconstruct ca 5,600 nucleotides from the virus genome, encompassing a part of the E1 protein gene (Figure 4).

ECSA: East Central South African.

The evolutionary history was inferred from nucleotide sequences using the neighbour-joining method and 500 bootstrap replications. Codon positions included were 1st plus 2nd plus 3rd. Non-coding positions were excluded from the analysis. All positions containing gaps and missing data were eliminated. There were in total 5,622 positions in the final dataset. Evolutionary analyses were conducted in MEGA7. Reconstructions using the Maximum Likelihood method provided a similar topology.

Sequence analysis indicated that the virus belongs to a previously described sub-lineage of the East Central South African (ECSA) lineage which includes isolates from the Central African region (Gabon, Republic of Congo) [2]). Similar to other members of this specific evolutionary group, the virus isolated from the index patient is carrying the E1-A226V mutation, which has been repeatedly associated with transmission by the vector *Aedes albopictus* [3]. The analysis of the full genome from the patient isolate is ongoing and the result will be submitted to GenBank.

#### Entomological results and control measures

Between 10 August and 11 September, the vector control operators implemented vector control measures in an area of 200 m radius around the index case’s home and workplace. Following the identification of new suspected cases, the treated area was extended to the whole neighbourhood (250 households) and to the areas visited by all cases while they were presumably viraemic.

*Bacillus thuringiensis israelensis* (Bti) was used for larvae and Deltaméthrin for adults. Larval breeding sites (rainwater storage tank, build elements, little containers) were treated with Bti or removed (spilled) when possible. Adulticide treatment was applied at the end of the night and sunrise with ultra-low volume (ULV) technics, twice (or more) on each target with minimal time interval of 2 days.

Entomological prospections, set up between 16 and 18 August after two of the seven adulticide sprayings, showed a medium density of adult mosquitoes and breeding sites in the neighbourhood. ‘Man-made’ breeding sites such as unprotected rainwater storage tanks, representing 50% of breeding sites, appeared to be the main driver of the mosquito densities. Adult mosquito traps showed a decrease of vector density: the mean number of mosquitoes caught in BG-GATs was 1.3 on 18 August and 0.2 on 6 September with a significant difference between the two dates (Dunn’s Multiple Comparison test, p value <0.05). These results suggest that the sprayings were effective in lowering mosquito density.

## Discussion

Since 2010, three emergences of chikungunya and six of dengue fever occurred in metropolitan France [4-9]. This outbreak with so far nine autochthonous chikungunya cases is the third occurrence of local transmission of CHIKV in France after that in Fréjus in 2010 and in Montpellier in 2014 [8,9]. The involvement of a CHIKV strain harbouring a mutation facilitating transmission by *Ae. albopictus* may explain the relative greater number of autochthonous cases observed in 2014 and 2017, compared with 2010.

Chikungunya is an arbovirus transmitted by *Aedes* mosquitoes, the main vector being *Ae. aegypti*. The 2006 epidemic of chikungunya in the South West Indian Ocean islands and subsequently in India and South-East Asia revealed that *Ae. albopictus* can act as an efficient vector of CHIKV [10,11]. This led to the implementation of enhanced arboviral surveillance during the time of vector activity from May to November, in the French departments where *Ae. albopictus* has been established. This surveillance relies on the mandatory notification of dengue, chikungunya and Zika virus disease cases, and on case finding through a laboratory network. When a case is detected, entomological investigations and, if necessary vector control measures, are implemented in each location visited by the case during their viraemic period [12].

Although up to 75% of chikungunya cases have classical symptoms of chikungunya [13], the primary case was not identified in the present outbreak. Thanks to previous sensitisation of GPs and biologists in the department Var, a region colonised by *Ae. albopictus*, the outbreak was detected early enough to timely implement vector control measures and to limit its spread. Nonetheless, in order to improve the reactivity of our surveillance system, regular awareness and training campaigns targeting GPs and medical laboratories, focusing on arbovirus diagnosis and reporting, should be conducted. Feedback to all those involved in the resolution of such outbreaks should further increase the acceptability of the system and, as a consequence, improve its efficiency.

Active case finding is still continuing and identified a cluster of two confirmed cases and five suspected cases in a town 10 km from Le Cannet-des-Maures. These cases are currently under investigation and there is an epidemiological link between them and le Cannet-des- Maures.

The present outbreak stresses the importance of long-term vector control in a densely populated residential area where *Ae. albopictus* is established. In the affected neighbourhood of Le Cannet-des-Maures, man-made mosquito breeding sites, in particular unprotected rainwater storage tanks, were most likely the main drivers of vector densities. Increases in dry periods due to annual climatic variations and possibly climate change on the long term, could lead to wider practice of rainwater storage in the summer. Thus, public awareness campaigns promoting good practices to limit the development of mosquito breeding sites should also focus on the use and appropriate maintenance of mosquito screens to protect water tanks.
